# The association of the 5-HTTLPR polymorphism and the response to different stressors in healthy males

**DOI:** 10.1007/s00702-021-02390-4

**Published:** 2021-08-10

**Authors:** Leandra Kuhn, Hannes Noack, Nadine Skoluda, Lisa Wagels, Ann-Kristin Röhr, Christina Schulte, Sana Eisenkolb, Vanessa Nieratschker, Birgit Derntl, Ute Habel

**Affiliations:** 1 Department of Psychiatry, Psychotherapy and Psychosomatics, Faculty of Medicine, RWTH Aachen, Pauwelsstrasse 30, 52074 Aachen, Germany; 2 Department of Psychiatry and Psychotherapy, Medical School, University of Tübingen, Tübingen, Germany; 3 Clinical Psychology of Adulthood, Faculty for Psychology, University of Vienna, Vienna, Austria; 4 Institute of Neuroscience and Medicine: JARA-Institute Brain Structure Function Relationship (INM 10), Research Center Jülich, Jülich, Germany; 5 Werner Reichardt Centre for Integrative Neuroscience, University of Tübingen, Tübingen, Germany; 6 Lead Research Network, University of Tübingen, Tübingen, Germany

**Keywords:** Stress, Serotonin, 5-HTTLPR, rs25531, Cortisol, Working memory, Response Inhibition

## Abstract

The experience of stress is related to individual wellbeing and vulnerability to psychopathology. Therefore, understanding the determinants of individual differences in stress reactivity is of great concern from a clinical perspective. The functional promotor polymorphism of the serotonin transporter gene (5-HTTLPR/rs25531) is such a factor, which has been linked to the acute stress response as well as the adverse effect of life stressors. In the present study, we compared the impact of two different stress induction protocols (Maastricht Acute Stress Test and ScanSTRESS) and the respective control conditions on affective ratings, salivary cortisol levels and cognitive performance. To this end, 156 healthy young males were tested and genotyped for the 5-HTTLPR/rs25531 polymorphism. While combined physiological and psychological stress in the MAST led to a greater cortisol increase compared to control conditions as well as the psychosocial ScanSTRESS, subjective stress ratings were highest in the ScanSTRESS condition. Stress induction in general affected working memory capacity but not response inhibition. Subjective stress was also influenced by 5-HTTLPR/rs25531 genotype with the high expression group showing lower stress ratings than lower expression groups. In line with previous research, we identified the low expression variant of the serotonin transporter gene as a risk factor for increased stress reactivity. While some dimensions of the human stress response may be stressor specific, cognitive outcomes such as working memory performance are influenced by stress in general. Different pathways of stress processing and possible underlying mechanisms are discussed.

## Introduction

Stress has wide-ranging effects on our everyday lives including cognitive functioning. The experience of acute stress activates our two prominent stress-response systems, the autonomic nervous system (ANS) and the hypothalamic–pituitary–adrenal axis (HPA) (Dickerson and Kemeny 2004; Foley and Kirschbaum 2010). These systems in turn affect a wide range of cognitive domains, such as memory and attention (Shields et al. 2019). In terms of working memory, Shields et al. found adverse effects of acute stress on working memory in their meta-analysis (Shields et al. 2016). Cortisol released by the HPA axis affects related brain structures such as the hippocampus and prefrontal cortex by binding to the glucocorticoid receptors (GR) and, thereby, impairing working memory performance (Arnsten 2009). Moreover, cortisol binds to mineralocorticoid receptors (MR) that have been linked to a rapid switch from cognitive to more habitual responses (Vogel et al. 2016). This switch promotes adapting to a stressful situation but may come at cost of higher cognitive functioning.

In the domain response inhibition, a less clear picture emerged in the meta-analysis (Shields et al. 2016). While no effect of acute stress on inhibition in general was found, results diverged in the sub-domains. Acute stress leads to impairments in cognitive inhibition (i.e., interference control). In contrast, an enhancing effect of stress was found for the sub-domain response inhibition (e.g., in the Stop Signal Task) which might be the result of improved motor control enabling individuals to withhold inappropriate responses (Chang et al. 2020). In their meta-analysis, the authors also suggested various situational factors, such as the delay between tasks, stress severity, and stressor type as potential task-specific moderators. At the level of interindividual differences only the effect of sex was taken into account providing evidence of a stronger stress effect on working memory in male compared to female participants. Other possible moderators such as genetic stress vulnerability and its interaction with different stressor types were not considered so far.

In this context, the serotonin transporter gene (*SLC6A4, 5-HTT*) gained much interest as a promising candidate gene. The short allele of the common promotor length polymorphism 5-HTTLPR is linked to reduced serotonergic functioning (Greenberg et al. 1999; Heils et al. 1997) and has been discussed as a risk factor for depression in interaction with stressful life events (Culverhouse et al. 2018; Karg et al. 2011; Risch 2009). However, recently, the largest meta-analysis on this topic so far concluded that 5-HTTLPR is not associated with depression (Border et al. 2019). The individual expression of the disorder with a unique set of symptoms caused by lots of conditional factors and situational triggers might cause variability. The development of depressive symptoms might, therefore, be too complex in nature to prove a direct influence of a single gene.

A more immediate and tangible influence of 5-HTTLPR is described by its association with the *acute* stress response. Acute stress can be induced in the laboratory under standardized conditions in a defined time frame leading to directly measurable outcomes. Thus, it serves as a circumscribed phenotype to investigate the influence of that polymorphism and related serotonergic functioning. Studies using standardized psychosocial stress protocols point towards a modulating role of 5-HTTLPR in the cortisol response. Several studies found a greater and more prolonged increase in salivary cortisol in S allele compared to L allele carriers (Dougherty et al. 2010; Gotlib et al. 2008; Way and Taylor 2010). However, other studies found no effect of 5-HTTLPR (Duman and Canli 2015; Wüst et al. 2009) and one even found higher cortisol levels in L allele carriers (Mueller et al. 2011). Li et al. (2019) found no effect on cortisol, but nervous mood as well as brain activity during memory performance under stress. Despite the inconsistency of single studies, a meta-analysis concluded that 5-HTTLPR has a small but significant effect on cortisol response with S allele carriers showing increased reactivity (Miller et al. 2013a, b). The biological mechanism behind this association is likely based on the vital role of serotonergic neurotransmission in acute HPA axis regulation (Porter et al. 2004). Animal studies suggest that 5-HTTLPR is even involved in persistent alterations in neural development of stress relevant cortico-limbic circuits (Murphy and Lesch 2008). The current study directly compares two operationalization methods in a large sample including a variant of the cold pressor task, a stress induction method that has not been included in the meta-analysis on 5-HTTLPR (Miller et al. 2013a). A single study found no effect of 5-HTTLPR on cortisol reactivity in a small sample although missing a cortisol increase in general, leaving room for further investigation (Markus and Firk 2009). Moreover, the meta-analysis did not take into account the single nucleotide polymorphism (SNP) rs25531 within the 5-HTT gene. Hu et al. found that the L allele with a common G substitution (L_G_) is functionally equivalent to S allele meaning lower serotonergic functioning (2006). Therefore, leaving out this information leads to a different categorization of individuals into presumably high risk or protective variants which could also have confounded the effects. This can be illustrated by the results of Alexander et al. (2014) who found more pronounced effects of the gene on cortisol reactivity after rs25531 was included in the analysis compared to the bi-allelic categorization.

Taken together, no study so far compared different stressors with regard to possible stressor-specific effects of 5-HTTLPR. Therefore, the present study can contribute to the understanding of the interaction of individual genetic vulnerability with different types of stressors. Moreover, genetic influences on subjective stress as well as further consequences of stress such as on cognitive performance remain unexplored up to now. Taking possible stress induced changes in working memory and response inhibition into account captures the stress response in a comprehensive way on the hormonal, subjective and cognitive level. The influence of 5-HTTLPR/rs25531 on these different outcomes can provide further insight into the role of the serotonergic system in all or just single facets of the stress response.

In the present study compares two rather new stress protocols. The Maastricht Acute Stress Test (MAST) combines the cold pressor test and psycho-evaluative elements (Smeets et al. 2012). The ScanSTRESS is a psychosocial stress task including a video-based social evaluative threat developed for fMRI experiments (Streit et al. 2014). Despite different foci of operationalization, both have proven to be effective. Importantly, both protocols hold the advantage of being adaptable for fMRI experiments. This possibility was of special concern, since the current study served as a predecessor study for a large-scale fMRI project on neural stress responses and influences of 5-HTTLPR. So far, only very little is known about the effects of 5-HTTLPR on rather physiological stressors, such as the cold pressor test that is part of the MAST. This comparison is also of interest, since most previous studies on 5-HTTLPR and acute stress were performed using the TSST that the ScanSTRESS is based on (Miller et al. 2013a). The current study, therefore, provides new insights into the generalizability or specificity of these genetic effects. In this context, the specific effects of both stressors on different outcome domains are first tested in the laboratory without the confounding anticipatory stress response elicited by the fMRI scanner. Especially for the ScanSTRESS, there is currently no data available on the mere effects of the paradigm without the scanner environment that causes stress by itself (Muehlhan et al. 2011).

In the current study, we operationalized stress reactivity as a multidimensional response, including cortisol, subjective stress and cognitive performance. We compared the potency of both stressors with regard to these dimensions. Moreover, we investigated whether an association of 5-HTTLPR/ rs25531 can be found with all three or just single dimensions and whether these associations are stressor-specific or generalizable. Based on previous evidence, we expect an increase from baseline to post stress in cortisol and subjective ratings compared to the respective control conditions. Regarding the direct comparison between stressors, we predict a more pronounced cortisol increase in the MAST compared to the ScanSTRESS condition due to its combination of physiological and psychological stress elements. Furthermore, we hypothesize that carriers of the 5-HTTLPR S allele show elevated stress responses compared to L homozygotes. Following the meta-analysis by Shields et al. (2016), we hypothesize a detrimental effect on working memory performance (n-back task) after stress compared to control conditions. We further expect enhanced response inhibition (Stop Signal Task) in both stress conditions compared to control conditions. The influence of stress on cognitive performance measures is assumed stronger for S allele carriers due to their presumably increased stress reactivity.

## Methods

### Participants

We recruited 156 healthy male participants at the universities of Aachen *(n* = 82) and Tübingen (*n* = 74) through public advertising. The number of participants per condition was balanced per site. Mean age was *M* = 23.56 years (*SD* = 3.97). Healthy males in an age range from 18 to 35 years were recruited. Only males were included in the present study to rule out menstrual cycle, i.e., fluctuating ovarian hormone levels, as a confounding factor that might interact with stress reactivity (Ossewaarde et al. 2010). Study eligibility was assessed in a semi-structured telephone interview and using the screening version of the German Structured Clinical Interview for Diagnostic and Statistical Manual of Mental Disorders (SCID, Wittchen et al. 1997). Exclusion criteria were current or lifetime psychiatric or neurological disorders, physical illnesses or any kind of medication that might affect activity of the HPA axis. Moreover, all participants were required to be non-smokers, of normal weight (17 < BMI < 30) and having a regular day–night cycle (i.e., no current shift working or jetlag). The study protocol was approved by the ethics committees of the medical faculty at the RWTH Aachen University and the University of Tübingen and was conducted in accordance with the declaration of Helsinki (World Medical Association 2013).

### Procedure

After a primary telephone screening, participants who fulfilled inclusion criteria were invited to a personal screening and a testing session on two different days. At the screening session, written consent was obtained followed by the structured clinical interview performed by a trained psychologist. Then, participants filled out psychometric tests measuring verbal intelligence and executive functioning followed by self-report questionnaires. Moreover, two nine ml EDTA tubes of venous blood were drawn at the screening session for later determination of the 5-HTTLPR variant of each participant.

To ensure that stress measures at the testing session were not affected by external confounding factors, participants were asked to refrain from physical exercise and alcohol consumption 1 day before the session. In addition, they were not allowed to consume caffeine or chewing gum at the testing day as well as food and drinks other than water one hour before study entry.

**Fig. 1:**
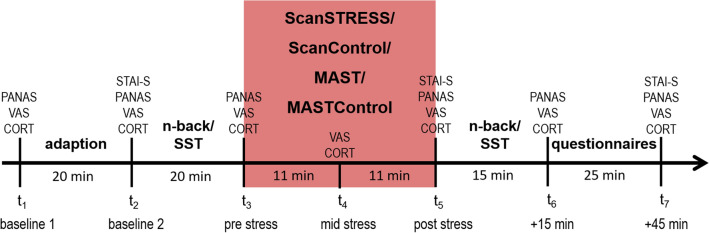
Procedure of the experiment. The order of the tasks as well as the timepoints of the different questionnaires (PANAS, STAI-S), the Visual Analogue Scale on subjective stress (VAS), and saliva samples for cortisol (CORT) are shown

To control for the diurnal variation of cortisol, all testing sessions started at either 1:30 pm or 5:00 pm, since basal cortisol secretion is rather low in the afternoon (Faiman and Winter 1971). Moreover, all sessions took place in the same testing room at both locations. On arrival, participants drank a glass of grape juice to control for blood sugar levels. After that, in 20-min adaption phase participants watched a documentary to alleviate potential stress responses caused by arriving in the laboratory situation. Then, participants completed the pre-session of either the n-back or the Stop Signal Task including prior instructions and practice trials. Subsequently, they underwent either the ScanSTRESS (Streit et al. 2014), MAST (Smeets et al. 2012) or one of the respective non-stress control conditions directly followed by the post session of the cognitive task. Both ScanSTRESS and MAST were implemented with Presentation^®^ Software (version 18.1, www.neurobs.com). Throughout the session, saliva samples for cortisol and affective ratings were obtained at seven timepoints, as shown in Fig. 1. For subjective ratings, the Positive and Negative Affect Schedule (PANAS; Watson et al. 1988), a Visual Analogue Scale from 0 to 100 on the current stress level as well as the State Trait Anxiety Inventory (STAI-S; Spielberger et al. 1983) should capture different facets of the affective stress response. After the last saliva sample that was taken 45 min after completion of the stress/non-stress protocol, participants were debriefed and received financial compensation.

### ScanSTRESS

ScanSTRESS is an achievement-oriented psychosocial stress paradigm specifically developed for fMRI experiments (Akdeniz et al. 2014; Streit et al. 2014). Inspired by the well-established TSST, it combines several stress-inducing components, such as pressure to perform, time pressure, forced failure, social-evaluative threat, uncontrollability and unpredictability. To test this new paradigm under normal laboratory conditions, we referred to the described procedure and stimuli with the exception that the participant was seated in front of a computer monitor in the laboratory instead of lying in the MRI tube.

The ScanSTRESS consists of control and stress blocks presented in a fixed alternating order. In the stress blocks, participants solved challenging mental rotation and arithmetic tasks under time pressure, while both, task speed and difficulty were adapted to the individual’s performance. In the control blocks, participants completed undemanding matching tasks without any time pressure. After instructions and practice trials, participants were introduced to an observer panel consisting of two trained researchers in professional attire. The participants were informed that, in the stress blocks, their behavior, mimics, and answers would be monitored by the panel via an online video transmission of their faces. For that purpose, a mock camera was installed on the participant’s monitor. A video transmission of the panel was presented to the participant during the task to induce social-evaluative threat. During stress blocks, the panel provided negative visual feedback and pressed a button on a buzzer—thereby sending a message to the participant’s screen indicating either an error or the instruction to respond faster. In control blocks, participants were informed that they would not be observed nor receive any feedback. The task was separated into two 11-min runs. During a short break between runs, the panel gave standardized negative verbal feedback about the previous performance telling the participants to try harder in the next run. The complete ScanSTRESS protocol took about 23 min.

### ScanControl

The non-stress control condition of the ScanSTRESS consisted of the control matching tasks only. To ensure a similar visual set up, a static image of a neutral jury panel not looking into the camera was shown during the task. Participants were told that this had nothing to do with the task and could be ignored. No panel was present und no feedback was provided. Apart from that, the procedure and durations were identical to the ScanSTRESS.

### Maastricht acute stress test

The MAST (Smeets et al. 2012) combines the physical stress of the Cold Pressor Test with challenging mental arithmetic elements under social evaluation as part of the TSST. In the original version, the MAST consists of a 5-min instruction and anticipation phase and a 10-min stress run. For better comparability with the ScanSTRESS, a short break before the second 10-min run was added. Altogether, the MAST took 25 min.

The task consists of time programmed instructions indicating different phases of the task. In the preparation phase, participants were seated in front of a computer monitor, where they received written instructions. Participants were told that they would be filmed by a camera installed at the monitor to analyze their facial expression throughout the task. To increase the credibility, they provided additional written consent. The task consisted of hand immersion trials and mental arithmetic trials presented in an alternating order. Participants were told that the duration of each trial had been randomly assigned by the computer but that ice water trials would not exceed 90 s and mental arithmetic trials would take at least 45 s. Actually, the paradigm terminated each trial after a fixed duration in the same order for all participants. In the hand immersion trials, participants had to immerse their non-dominant hand, including the wrist, into a bucket of ice-cold water (2 °C) as long as the instruction appeared on the monitor. When the trial was terminated participants placed their arm on a towel and the mental arithmetic trial started. In the mental arithmetic trials, participants counted backwards in steps of 17 from a three- or four-digit number as fast and accurate as possible until the next ice water trial started. In case of a mistake they had to start again from the beginning. The procedure was supervised by an additional experimenter who was present only for this part of the procedure and pointed out mistakes without giving any positive feedback.

### MASTControl

The non-stress MASTControl included hand immersion trials with luke-warm water (35–37 °C) and counting trials in between. In the counting trials, participants were asked to count from 1 to 25 at their own pace and start again when finished until the next hand immersion trial started. An additional experimenter was present but did not evaluate the performance. Apart from that, the overall procedure and durations were identical to the MAST.

### N-back

The n-back is a standard working memory (WM) task in which participants have to memorize a sequence of letters and indicate whether the current letter was already presented *n* trials back (Kirchner 1958). Participants completed the letter version in two difficulty levels—the 2- and 3-back WM load. Always starting with the 2-back load, the n-back was performed both pre and post the stress or control conditions. Participants responded via button press with the right index finger if the current letter appeared two/three trials before and with the left index finger if it did not. They were asked to answer as fast and as accurately as possible. Before the pre run started, participants were instructed and completed practice trials including feedback for each WM load. The two WM loads were presented in separate runs starting with the 2-back load. For the 2-back load letters were presented in four blocks including 26 trials each encompassing 104 trials. In the subsequent 3-back run encompassing 108 trials, four blocks of 27 trials each were presented. Letters were presented in white on a black background for 500 ms followed by a black screen. The total trial duration was 3000 ms. Targets appeared in about 33% of the trials.

### Stop signal task

The Stop Signal Task (SST) is a widely used measure of response inhibition in which participants have to withhold a predominant motor response given a certain stop signal (Logan 1994). In the current study, a GO-Trial started with a white fixation circle presented on a black background screen. After 500 ms, a white arrow appeared in the circle pointing right or left in a pseudorandomized order. Participants had to indicate the direction of the arrow via button press as fast as possible. After button press or a maximal trial length of 1 s, the screen turned black until a new trial started. In about 33% of the trials, the white circle turned blue indicating a stop signal so participants should inhibit the button press. The stop signal occurred after a pre-specified Stop Signal Delay (SSD) that was varied according to a staircase procedure (Aron and Poldrack 2006). Therefore, one of four independent staircases for dynamic adaptation of the SSD was assigned to each stop trial in a pseudorandomized order. Staircases started at a SSD of either 100, 150, 200, or 250 ms and, after that adapted to the participants’ performance. In case of a successful inhibition, the SSD was increased by 50 ms in the next stop trial of that staircase thereby increasing the difficulty. In contrast, if the participant did not inhibit the response in a stop trial the next SSD of that staircase decreased by 50 ms facilitating inhibition. Different starting points and the pseudorandomized assignment of Stop Trials to the different staircases was used to avoid habituation effects. This way, a mean inhibition rate of approximately 50% was ensured by the end of the experiment. In total, the SST consisted of 384 trials which included 128 stop trials.

### Cortisol

Saliva samples were taken at seven timepoints during the testing session (see Fig. 1) using Cortisol Salivettes® (Sarstedt, Nürnbrecht, Germany). We obtained a first baseline sample ca. 10 min after arrival and a second baseline sample after another 20-min adaptation phase, then, one directly before the stress or control protocol, in the short break in the middle of the stress /control protocol and directly after. Another saliva sample was taken after the post run of the cognitive task (about 15 min after cessation of stress/control protocol) and the last one 45 min after the stress/control protocol ended to capture the regeneration phase adequately. To collect saliva, participants put a synthetic fiber swab in the mouth for 1 min, allowing to absorb secreted saliva. Participants were instructed not to chew on the swab to avoid tactile stimulation which in turn is known to increase saliva flow rate. All samples were stored at  − 20 °C until they were sent to the laboratory for analysis. For cortisol analysis, salivettes were thawed and centrifuged for 2 min at 1000 rpm to collect saliva. Further analysis was performed using an enzyme immunoassay (IBL International, Hamburg, Germany, Cortisol ELISA, REF RE52611) according to the manufacturer’s instructions. Average cortisol levels were taken from duplicate runs if intra-assay variation was below 10%.

### Genotyping

EDTA blood samples were stored at  − 80 °C until analysis. Genomic DNA from peripheral lymphocytes was extracted using the QIAamp Blood Maxi Kit (Qiagen, Hilden, Germany), and quality and quantity was measured and controlled using a Qubit^®^ 2.0 Fluorometer (life technologies, Darmstadt, Germany) and Qbit Assays for dsDNA following the manufacturer’s instructions. The 5-HTTLPR locus was amplified by PCR following the protocol of Lesch et al. (1996) with slight modifications. In brief, PCR was carried out in a 25 µl reaction volume containing 20 ng of genomic DNA and 0.4 µM of each oligonucleotide primer (forward: 5′-GGC GTT GCC GCT CTG AAT GC-3′ and reverse: 5′-GAG GGA CTG AGC TGG ACA ACC AC -3′) using the GoTaq^®^ Colourless Master Mix (Promega, Mannheim, Germany). Following heat denaturation of the samples (3 min at 95 °C), 35 cycles were carried out consisting of 30 s at 95 °C, 30 s at 63 °C and 1 min at 72 °C, followed by a final extension step of 10 min at 72 °C. PCR products were resolved on a 2% agarose gel and visualized under UV illumination using peqGREEN (1:20,000; Peqlab, Erlangen, Germany). Accuracy was assessed by duplicating 15% of the original sample, and reproducibility was 100%. Genotyping of rs25531 has been performed by digesting 10 µl of the PCR product over night at 37 °C with 0.1 µl MSP1 (New England Biolabs, Ipswich, MA, USA) and 1 μl buffer per sample and running it on an 4% agarose gel for 120 min at 180 V. 15% of the samples have been run in duplicates and reproducibility was 100%. Genotype calls have been made by two individuals independent of each other and concordance was 100%.

As the L_G_ variant has been shown to be functionally equivalent to the S allele, 5-HTTLPR/rs25531 groups were defined accordingly: the low expression group consisted of the combinations S_A_/S_A_, S_A_/L_G_, and L_G_/L_G_; the intermediate expression group comprised the S_A_/L_A_ and L_A_/L_G_ variants, and the high expression group referred to L_A_/L_A_ homozygotes. The distribution of 5-HTTLPR/rs25531 groups was as follows: *n* = 31 low expression, *n* = 81 intermediate expression, *n* = 39 high expression. The genotype frequencies did not deviate from Hardy–Weinberg equilibrium (*p* = 0.609).

### Statistical analyses

To evaluate the effectiveness of the different stress protocols, we compared changes in cortisol levels and VAS stress ratings as two indicators of stress reactivity. Therefore, we ran two repeated measures analyses of variance (ANOVA) including the within-subjects factor timepoint (1–7) as well as the between-subject factors condition (stress vs. control) and method (ScanSTRESS vs. MAST) on our primary outcomes, the cortisol levels and the VAS subjective stress ratings. As the cortisol data were positively skewed, a natural logarithm (ln) transformation was performed leading to a normal distribution at every timepoint confirmed by Kolmogorov–Smirnov Test (all *p*’s > 0.051). For subsequent analyses, ln-transformed data were used, whereas non-transformed units were used for figures. For descriptive purposes, we also calculated the percentage of cortisol responders characterized by a baseline to peak increase of at least 1.5 nmol (≙ 0.054 µg/dl) referring to the responder criterion proposed by Miller et al. (2013b). Similarly, we used rmANOVAs on the effect of time, condition and method on secondary outcomes, such as the positive and negative affect scale of the PANAS and the STAI but with fewer timepoints (six for the PANAS and three for the STAI). When sphericity assumptions were violated, Greenhouse–Geisser corrected *p* values are reported.

Furthermore, using univariate ANOVAS, we looked for an effect of 5-HTTLPR/rs25531 (high, intermediate, low expression) on our main outcome, cortisol levels, and subjective VAS ratings calculated as areas under the curve (AUC; Pruessner et al. 2003) to test if one subgroup is more vulnerable for acute stress (5-HTTLPR/rs25531 × condition × method).

The primary outcome variable for the n-back task was the parameter d’, which is the difference of the z-transformed number of hits and the z-transformed number of false alarms. D’ was calculated for each working memory load (2- and 3-back) and timepoint (pre and post).

Outcome variable in the SST was the Stop Signal Reaction Time (SSRT) as an estimate of the duration of the stop process. We applied the mean method based on the horse race model (Verbruggen and Logan 2009). For each participant, the mean SSD was subtracted from the median RT of all correct go trials.

For the n-back, a three-way rmANOVA including the factors timepoint (pre vs. post), condition (stress vs. control) and method (ScanSTRESS vs. MAST) was conducted. We proceeded the same way with the SSRT. In addition, we performed separate ANOVAS including the factors 5-HTTLPR/rs25531 (high, intermediate, low expression) and cortisol responder group (responder vs. non-responder). These analyses were done on an exploratory basis, since the further division of each the SST and n-back sample led to very small subgroups.

Alpha level was set at 0.05 and Bonferroni adjustments for multiple comparisons were used for post-hoc t-Tests tests. For detailed tables of the ANOVAS and post-hoc tests see supplementary material.

### Missing data

For cortisol analysis, two subjects were excluded due to extreme values and two did not have a complete set of cortisol samples. Subjective ratings of four participants were not obtained for all timepoints, so they were excluded from VAS analysis. Genetic data was available for 147 participants. The distribution of the sample to the different conditions for the different analyses are summarized in Table 1.

**Table 1:** Sample sizes for the different outcome variables, genetic variants and cortisol responders for the reported analyses.

	ScanSTRESS	ScanControl	MAST	MASTControl
Outcome variables				
Cortisol	39	34	38	39
VAS	38	35	39	40
n-back	19	17	19	13
SST	19	17	20	17
5-HTTLPR variant				
Low expression	9	6	8	8
Intermediate expression	20	19	23	19
High expression	10	10	7	12
Cortisol responder	10		22	
In n-back group	6		11	

## Results

### Cortisol

**Fig. 2:**
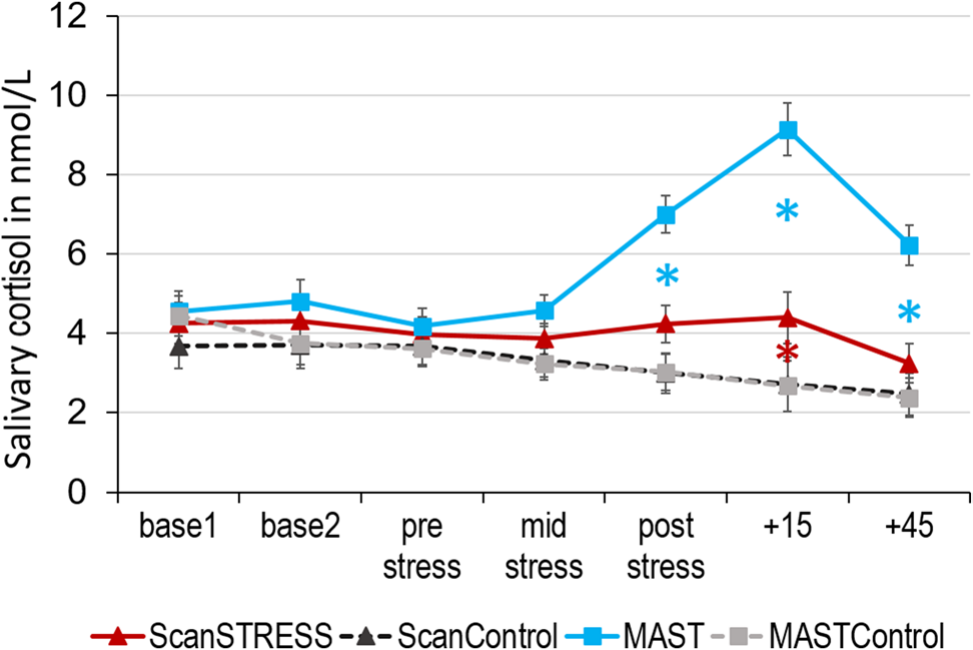
Mean salivary cortisol levels in nmol/L for the different conditions at the seven different timepoints. Error bars reflect standard error of mean

Results on the salivary cortisol response are displayed in Fig. 2. The rmANOVA revealed significant main effects of time [*F*(2.74, 400.53) = 7.71, *p* < 0.001, *η*_*p*_^*2*^ = 0.05], condition [*F*(1,146) = 12.27, *p* = 0.001, *η*_*p*_^*2*^ = 0.08] and method [*F*(1,146) = 6.87, *p* = 0.01, *η*_*p*_^*2*^ = 0.05] as well as a time x condition x method interaction [*F*(2.74, 400.53) = 5.81, *p* = 0.001, *η*_*p*_^*2*^ = 0.04]. Post-hoc analyses showed the greatest cortisol increase in the MAST condition, which was significantly higher compared to MASTControl at the timepoints mid (*p* = 0.015) and after stress as well as + 15 and + 45 min (all *p*’s < 0.001). In addition, the cortisol increase in the MAST group was higher than in the ScanSTRESS group at these timepoints (mid stress: *p* = 0.04, post stress, + 15 min and + 45 min: all *p*’s < 0.001). In the ScanSTRESS group, there was a significant increase in cortisol at 15 min after stress compared to the ScanControl condition (*p* = 0.008). For additional information on post-hoc tests see supplementary material. The percentage of cortisol responders was 57.9% in the MAST and 25.6% in the ScanSTRESS condition.

### Subjective stress ratings

**Fig. 3:**
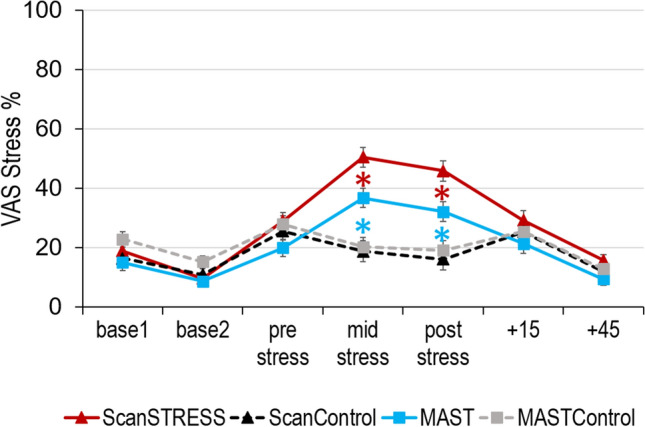
Subjective stress ratings in the Visual Analogue Scale (VAS) in percent corresponding to millimeters on the scale. Mean values are presented for each condition at each timepoint. Error bars reflect standard error of mean

For the subjective stress ratings in the VAS, the rmANOVA revealed a significant main effect of time [*F*(3.82, 148) = 69.76, *p* < 0.001, *η*_*p*_^*2*^ = 0.32] and condition [*F*(1, 148) = 5.33, *p* = 0.022, *η*_*p*_^*2*^ = 0.04] as well as interaction effects of time x condition [*F*(3.82, 148) = 40.33, *p* < 0.001, *η*_*p*_^*2*^ = 0.21], time × method [*F*(3.82, 148) = 2.60, *p* = 0.017, *η*_*p*_^*2*^ = 0.02] and condition × method [*F*(1, 148) = 5.46, *p* = 0.021, *η*_*p*_^*2*^ = 0.04] visualized in Fig. 3. Here, both stress methods provoked higher ratings than the respective control conditions in the middle of the two stress runs (ScanSTRESS: *p* < 0.001; MAST: *p* < 0.001) and directly after stress (ScanSTRESS: *p* < 0.001; MAST: *p* = 0.006). Moreover, the ScanSTRESS resulted in higher subjective stress rating than the MAST mid and after stress (both *p*’s < 0.01) as well as + 45 min (*p* = 0.026). The results of the PANAS and STAI ratings are reported in the supplementary material.

## 5-HTTLPR/rs25531

**Fig. 4:**
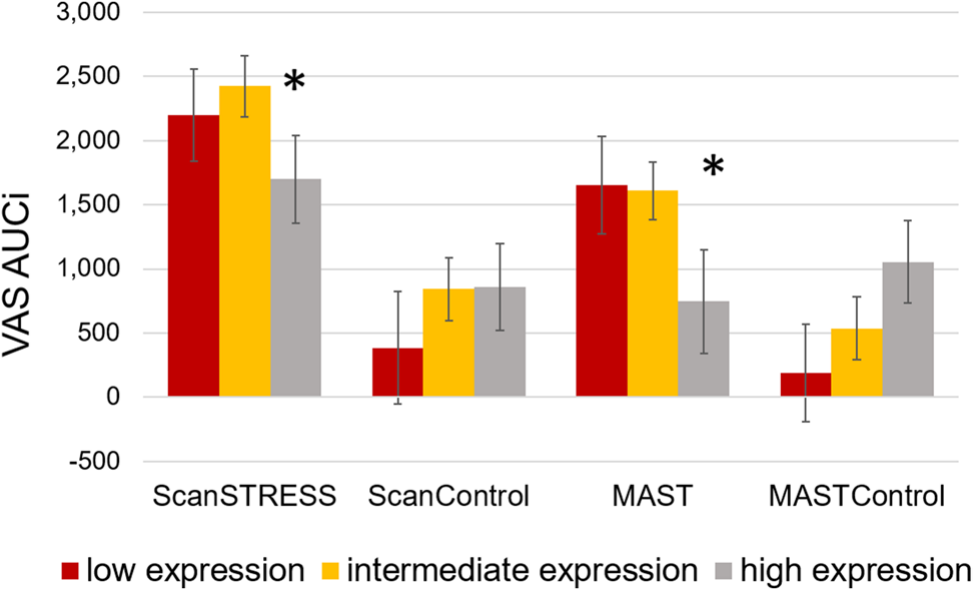
Increase of subjective stress ratings in the VAS as area under the curve for each condition and genotype. Error bars reflect standard error of mean

To investigate the association of the 5-HTTLPR*/rs*25531 genotype with the cortisol response, we calculated the area under the curve (AUCi) as an index for cortisol secretion. A univariate ANOVA showed a main effect of condition [*F*(1,138) = 9.13, *p* = 0.003, *η*_*p*_^*2*^ = 0.06] and method [*F*(1,138) = 4.0, *p* = 0.048, *η*_*p*_^*2*^ = 0.03] but no interaction with 5-HTTLPR*/rs*25531 (*p*’s > 0.10). For subjective stress ratings in the VAS, a main effect of condition [*F*(1,138) = 31.32, *p* < 0.001, *η*_*p*_^*2*^ = 0.19] and method [*F*(1,138) = 5.13, *p* = 0.025, *η*_*p*_^*2*^ = 0.04] emerged. Moreover, an interaction effect of condition and 5-HTTLPR*/rs*25531 was found [*F*(2,138) = 4.19, *p* = 0.017, *η*_*p*_^*2*^ = 0.06] and is depicted in Fig. 4. Only after stress, the high expression group showed a smaller increase in stress ratings than the other groups (p = 0.037, *η*_*p*_^*2*^ = 0.05).

### Cognitive performance

Concerning the WM performance in the n-back, a significant improvement of performance from pre to post was observed. For the 2-back, no effects of time, condition or method on d’ scores arose. Only for the 3-back, a trend wise interaction of time x condition was observed [*F*(1,64) = 3.39, *p* = 0.070, *η*_*p*_^*2*^ = 0.05]. Post-hoc tests revealed a significant improvement in performance from pre to post (ScanControl: *p* = 0.021, MASTControl: *p* = 0.025) that was absent in both stress conditions (ScanSTRESS: *p* = 0.980, MAST: *p* = 0.198).

**Fig. 5:**
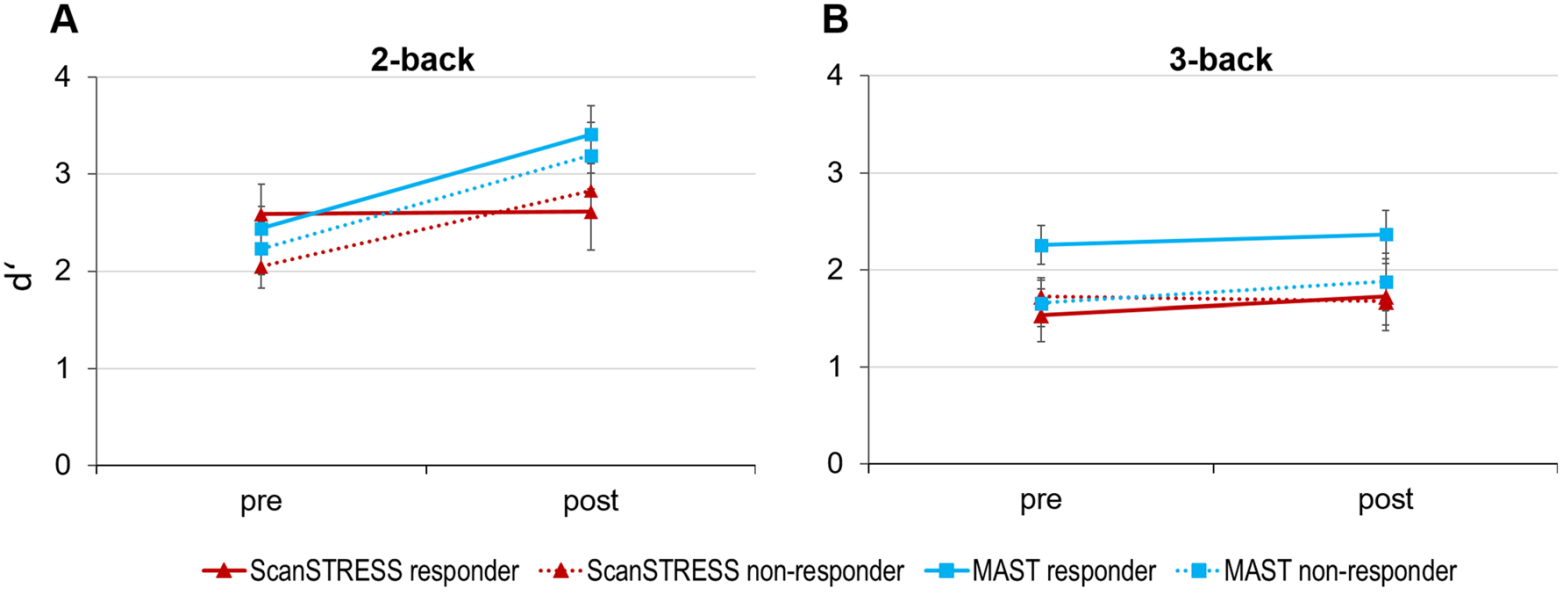
**A** Working memory performance in the 2-back WM load measured by d’ from pre to post stress. Means for both stress conditions depending on the cortisol responder group are depicted. **B** Mean d’ values for the 3-back WM load. Error bars reflect standard error of mean

Given our hypothesis that especially highly stress reactive individuals exhibit changes in cognitive performance, we explored whether we would find effects in subgroups characterized by cortisol response or 5-HTTLPR*/rs*25531 genotype. Indeed, we found an interaction effect of condition and responder group on d’ difference scores [*F*(3,59) = 2.82, *p* = 0.047, *η*_*p*_^*2*^ = 0.13] visualized in Fig. 4. In the 2-back WM load, only in the ScanSTRESS condition, cortisol responders differed significantly from non-responders (*p* = 0.025). Unlike non-responders, responders did not improve their WM performance from pre to post stress (Fig. 5). In the MAST condition, all participants improved their performance regardless of their cortisol response. No effects of 5-HTTLPR/rs25531 on WM performance were observed.

Response inhibition, measured by the SSRT, was influenced neither by condition or method, nor by 5-HTTLPR/rs25531 or responder group (all *p*’s > 0.12).

## Discussion

The aims of the present study were twofold. First, we wanted to directly compare two different stress protocols that are versatile and potentially suitable for further fMRI studies regarding cortisol and affective reactivity as well as subsequent cognitive performance. These results contribute to a better understanding of stressor-specific effects on physiological and subjective reactivity as well as cognitive performance. Second, we addressed genetic differences that might influence stress reactivity in general or in response to specific stressors. Using a multidimensional approach, we observed (a) both stress induction methods were successful in eliciting a stress response compared to the respective control conditions including working memory performance, and (b) a stressor-specific response pattern emerged. While the MAST elicited the strongest salivary cortisol response, the ScanSTRESS induced higher subjective stress ratings. Concerning 5-HTTLPR/rs25531, the protective high expression variant was associated with lower subjective stress, while cortisol levels were not affected by genotype. Regarding cognitive performance, stress in general affected working memory with a high load but not response inhibition. There was also a hint towards an interaction of stressor and cortisol response in the 2-back WM task, with cortisol responders in the ScanSTRESS condition showing a diminished training effect that was detectable in all other groups. Although small subgroup sizes demand a cautious interpretation, this points towards a combination of stressor-specific and individual-specific characteristics to explain stress reactivity.

Concerning our primary outcome variable, the increase in salivary cortisol levels, the superiority of the MAST is in line with previous literature. While the MAST provoked reliable hormonal changes in several studies (Meyer et al. 2013; Quaedflieg et al. 2015; Smeets et al. 2012), the ScanSTRESS failed to do so in the original sample (Streit et al. 2014). This difference is also evident regarding the previously reported cortisol responder rate, which is 85% for the MAST (Smeets et al. 2012) and 52% for the ScanSTRESS (Streit et al. 2014) the former being comparable to the TSST (Miller et al. 2013b). A possible explanation for the superiority of the MAST derives from the advantage of combining physical stress caused by repeatedly being exposed to cold pain and psychological stress induced by social-evaluative math performance task. Such a threat to physical integrity requires an immediate bodily response that provides us with necessary resources to fight or flight. Therefore, physical stressors have been presumed to directly trigger the HPA axis through a subcortical pathway including the brainstem and hypothalamus (Ulrich-Lai and Herman 2009). In contrast, solely psychosocial stressors involve more indirect pathways through the forebrain and, thereby, are capable of activating the HPA axis as well but do not possess this biological immediacy (Herman et al. 2003). This indirect pathway might, on the other hand, preferably trigger other processes such as affective changes that need higher order processing.

The fact that the ScanSTRESS does not produce as reliable cortisol increases as the also psychosocial TSST could be the result of the adaptions for the fMRI environment. These adaptions cause a more indirect interaction via the computer. In the TSST, participants face the physically present jury panel directly in a mock job interview. In contrast, in the ScanSTRESS, participants are spatially separated from the jury panel interacting only virtually which has been shown to diminish cortisol response (Helminen et al. 2019). Moreover, the repeated control phases, that are important for fMRI analyses, might allow the body to recover in between stress phases, while the whole ScanSTRESS might still be experienced as subjectively stressful.

The more pronounced affective changes in the ScanSTRESS condition might be due to the predominant social evaluative threat induced by the two-headed jury panel that gives constant visual negative feedback supported by a destructive verbal feedback in between runs. Such social information is usually processed via a pathway including the medial prefrontal cortex and the limbic system and it is interpreted according to prior experiences (Herman et al. 2005). Thereby, the ScanSTRESS might be rather attributed and subsequently rated as “stress” as we experience it nowadays—solving difficult tasks under time pressure while being observed and evaluated by others. In comparison, physiological stress in the MAST could be rather attributed as pain or discomfort. To cope with the demands of the ScanSTRESS, psychological rather than bodily resources need to be released. Therefore, it makes sense for the individual to determine the situation as stressful without a full preparatory fight and flight response. Nevertheless, the pronounced subjective stress reaction deserves as much attention as cortisol changes, since these changes are presumably crucial for individual well-being. Influential stress models such as Lazarus’ transactional stress model mainly focus on subjective interpretation of stressors (Lambert and Lazarus, 1970). In this context, the ScanSTRESS seems to be experienced as more stressful than the MAST. In addition, our results highlight the importance of the situation in the fMRI scanner as an effective component of the ScanSTRESS. This component appears to be decisive for the cortisol response.

The present study was the first to directly compare these two different stress protocols. Moreover, our results provide a benchmark for the affective stress response in the ScanSTRESS, since previous studies mostly focused on brain activation and physiological parameters and only one included emotional stress ratings (Akdeniz et al. 2014). To investigate the different pathway hypothesis of physical vs. psychosocial stressors, further neuroimaging studies are indispensable. These could easily make use of the fMRI suitability of the ScanSTRESS and compare it to an already adapted version of the MAST, the iMAST (Quaedflieg et al. 2013).

So far, our study was the first to investigate the impact of 5-HTTLPR/rs25531 across two different stress induction methods. Our findings are in line with previous studies identifying the lower expression groups as more reactive to stress. However, these studies mainly focus on cortisol response and did not report influences on subjective stress. In contrast, 5-HTTLPR/rs25531 did not affect cortisol increase in the present study. This could be due to the rather small cortisol effect in the ScanSTRESS. This may indicate that the genetic influence on cortisol reactivity is not generalizable across different stressors, while the effect on subjective ratings seems rather robust.

These results point towards acute subjective stress responses as a promising indicator in the context of stress-related mental disorders, such as depression. Putatively, individuals with lower expression variants of 5-HTTLPR/rs25531 are more susceptible to different forms of stress and respond with elevated subjective responses. This way, stress may more likely exceed individual coping abilities in these individuals with a genetic vulnerability, possibly resulting in more mental symptoms (e.g., depressive or anxious mood, anhedonia or reduced motivation) and health problems.

Concerning cognitive performance, a general stress effect emerged only in the working memory domain. At a high WM load, both stress inductions prevented a training effect that was observed in the control groups from pre to post stress. That this effect is only observable in the high load and not in the low load condition of the task is in line with previous literature (Oei et al. 2006). Stress might especially deteriorate higher cognitive functions as these require most resources. A basic level of working memory and learning is important to cope with the situation while solving complicated tasks is less relevant in face of a stressor. The rising cortisol with its peak directly after the cognitive performance might have interfered with hippocampal and prefrontal activity that is crucial for working memory. The critical role of cortisol is further emphasized by our explorative results after dividing the group into cortisol responders and non-responders. For the 2-back WM load, cortisol responders did not show a training effect that was evident in the other groups. Interestingly, these differences became evident after the ScanSTRESS that resulted in an overall weaker cortisol response. Possibly, individuals that still show a hormonal reaction are especially vulnerable to stress that is reflected in working memory problems.

### Limitations

Although our sample size can be considered large compared to other behavioral studies, it was still not sufficient to tackle all possible interactions of the different stressors, responder groups and genotype. The unequal distribution of the 5-HTTLPR variants resulted in rather small groups of the presumably high-risk low expressive and protective high expressive genotype groups. In combination with the rather complex experimental design, comparing multiple conditions resulted in small cell sizes. In particular, the statistical power to test paradigm-by-genotype interactions is quite low. Results involving genetic information should, therefore, be interpreted cautiously.

Moreover, the candidate gene approach has been discussed in the recent years. Technological advances such as genome-wide association studies (GWAS) gave more reliable insight into genetic risks for mental disorders and qualified the importance of single genes. In this context, the association of 5-HTTLPR and depression was challenged, and the risk of false positive results was emphasized. However, the GWAS approach requires large sample sizes that are beyond reach in experimental designs including different methods and stress measures so far. Nevertheless, interaction between experimental conditions and genetic influences are still a relevant research question with the advantage of being able to address questions in a more causal way. In addition, candidate genes are still necessary to explore the exact function of a gene of interest. Regarding 5-HTTLPR, its importance for serotonergic functioning gives reason to hypothesize an association with stress reactivity in light of the wide-ranging effects of serotonin on the HPA-axis and mood in general. Yet, our single candidate gene approach does not account for possible interactions of different genes.

Our sample is also limited to young males who are mostly students. This reduces generalizability, since sex differences in stress responses have often been found in previous research (Kudielka and Kirschbaum 2005). Moreover, our sample is characterized by a high level of education and is probably used to perform under pressure. On the one hand, this underpins our findings that both protocols still induce a stress response in these individuals. On the other hand, this might, in part, explain the absent effects on cognitive performance. A more representative sample with different educational backgrounds might increase the potential to find stress effects on cognitive performance. Besides, the restriction to only healthy participants without psychiatric symptoms precludes a direct link to mental disorders or evaluations whether the observed stress reactions are a risk factor or an adaptive response.

Our study design, including a pre and post measure of cognitive performance, bears the problem of training effects. Being already familiar with a task possibly interferes with stress effects. Furthermore, it is possible that the n-back, including the challenging 3-back WM load, might induce pressure to perform by itself and, therefore, confound with stress effects. These potential drawbacks could be addressed with within-subjects crossover designs, which, however, bring about other problems, such as order effects.

Finally, by measuring cortisol levels, only the adrenal output of the HPA axis is considered. Processes *during* stress can be revealed by neuroimaging studies or by measuring other physiological parameters reflecting the activity autonomic nervous system, such as electrodermal activity, heart rate or alpha-amylase (Nater and Rohleder 2009).

### Conclusion

Our study was the first to directly compare the MAST and the ScanSTRESS with respect to the multidimensional stress response including two domains of executive functioning. While the MAST led to a superior cortisol response, the ScanSTRESS provoked higher subjective stress ratings. Working memory was partly deteriorated by stress, whereas response inhibition remained unaffected. Regarding the 5-HTTLPR/rs25531 polymorphism, the results fit to previous findings identifying the lower expression variants as a risk factor for increased stress reactivity, yet only on the subjective and not on the hormonal level. Future studies could disentangle the specificity of the stress response and genetic influences by systematically varying different kinds of stressors in large samples. Beyond that, neuroimaging studies comparing different kinds of stressors can provide insight into the underlying neural mechanisms. Our results point towards different stress processing pathways that are differentially affected by genetic influences that should be investigated further.

## Data Availability

The data sets generated during and/or analyzed during the current study are not publicly available, since making the data publicly available would contradict the agreement with the local ethics committee. Data sets are available from the corresponding author on reasonable request.
